# Quality in Assisted Living: Does It Lie in the Eyes of the Beholder?

**DOI:** 10.1093/geroni/igaa057.2578

**Published:** 2020-12-16

**Authors:** Diana White, Tunalilar Ozcan, Serena Hasworth, Jaclyn Winfree

**Affiliations:** Portland State University, Portland, Oregon, United States

## Abstract

Quality is defined in multiple ways and by different stakeholders (e.g., residents, regulators, informed observers). Using a two-stage stratified sampling strategy, we collected data from N=241 residents living in 31 assisted living and residential care communities (AL/RC) in Oregon. Residents rated their overall satisfaction and satisfaction with the AL/RC as a place to live and to receive care. Each interviewer completed a facility profile summarizing their observations about the setting, including quality of staff-resident interactions and physical environment. Residents and interviewers were also asked whether they would recommend the community to others. Finally, we used deficiency citations given during regular inspections by the licensing agency to proxy regulatory perspective. Results show that perceived quality varied by stakeholder (e.g., residents’ assessments differed from deficiency citations). Given this variation, findings suggest that efforts to make quality indicators publicly available should include multiple measures and perspectives, especially residents.

